# Prevalence and predictors of workplace violence against emergency physicians in China: a cross-sectional study

**DOI:** 10.1186/s12960-022-00784-3

**Published:** 2023-02-08

**Authors:** Shijiao Yan, Jing Feng, Yong Gan, Rixing Wang, Xingyue Song, Zhiqian Luo, Xiaotong Han, Chuanzhu Lv

**Affiliations:** 1 Department of Emergency Medicine, Hunan Provincial People’s Hospital/The First Affiliated Hospital, Hunan Normal University, Changsha, Hunan China; 2 School of Public Health, Hainan Medical University, Haikou, Hainan China; 3 Department of Social Medicine and Health Management, School of Public Health, Tongji Medical College, Huazhong University of Science and Technology, Wuhan, Hubei China; 4 Department of Emergency, Hainan Clinical Research Center for Acute and Critical Diseases, The Second Affiliated Hospital of Hainan Medical University, Haikou, Hainan China; 5 Research Unit of Island Emergency Medicine, Chinese Academy of Medical Sciences (No. 2019RU013), Hainan Medical University, Haikou, Hainan China; 6 Emergency and Trauma College, Hainan Medical University, Haikou, Hainan China; 7 Clinical Research Center for Emergency and Critical Care in Hunan Province, Changsha, Hunan China; 8 Emergency Medicine Center, Sichuan Provincial People’s Hospital, University of Electronic Science and Technology of China, No. 32 Yi Huan Lu Xi Er Duan, Chengdu, 610072 Sichuan China; 9 Key Laboratory of Emergency and Trauma of Ministry of Education, Hainan Medical University, Haikou, Hainan China

**Keywords:** Workplace violence, Emergency physicians, Epidemiology, Prevalence, China

## Abstract

**Background:**

Workplace violence (WPV) is considered a global problem, particularly in the health sector; however, no studies have assessed the national prevalence of WPV against emergency physicians and the associated factors in China.

**Methods:**

A national cross-sectional survey was conducted in 31 provinces/autonomous regions/municipalities across China between July 2019 and September 2019. A total of 15 455 emergency physicians were selected using a multistage stratified random sampling method. A structured self-administered questionnaire was used to collect information on WPV and potential associated factors among emergency physicians. Descriptive and multivariable logistic regression analyses were used to identify the predictors of WPV.

**Results:**

A total of 14 848 emergency physicians responded effectively (effective response rate: 96.07%). Of the respondents, 90.40%, 51.45%, and 90.00% reported exposure to any type of WPV, physical or nonphysical violence in the preceding year, respectively. Verbal aggression (87.25%) was the most common form of violence, followed by threat (71.09%), physical assault (48.24%), verbal sexual harassment (38.13%), and sexual assault (19.37%). Patients’ families were the main perpetrators of these incidents. Unmet patient needs, taking drugs or drinking, and long waiting times were the main contributors to WPV. Physicians who were from low-developed regions, female, and without shift work were less likely to have experienced any type of WPV. Chinese emergency physicians who were from medium-developed regions, had a bachelor’s degree, worked in a higher level hospital, had a higher professional title, with lower incomes, had a history of hypertension or coronary heart disease, were smokers or drinkers, and worked in hospitals without preventive measures or training for WPV and not encouraging to report WPV were more likely to have experienced any type of WPV. The predictors of WPV varied in different types of WPV.

**Conclusions:**

This study shows that the prevalence of WPV against emergency physicians is high in China. Measures should be taken at the physicians, patients, hospital, and national levels to protect GPs from WPV; for example, improving physicians’ level of service and hospital’ reporting procedures. Creating a prevention strategy and providing a safer workplace environment for emergency physicians should be prioritized.

**Supplementary Information:**

The online version contains supplementary material available at 10.1186/s12960-022-00784-3.

## Background

Workplace violence (WPV) is defined as “incidents where staff are abused, threatened, or assaulted in circumstances related to their work, including commuting to and from work, involving an explicit or implicit challenge to their safety, well-being, or health” [1]. WPV against healthcare workers (HCWs) has become an important global challenge. A meta-analysis of WPV for all HCWs identified that 61.9% of HCWs reported exposure to any form of WPV [2]. WPV may contribute to reduced job satisfaction, commitment and efficiency, poor quality of life, increased stress, sleep disruption, burnout, turnover, and even death [3–9]. Therefore, the primary prevention of such violence worldwide is an important public health priority.

Violence against HCWs has been increasingly reported in China. The overall prevalence of WPV against healthcare professionals was 62.4%, and the prevalence of WPV across different specialties was significantly different, with WPV being highest in departments of emergency medicine (79.8%), followed by departments of pediatrics (73.7%), surgery (62.4%), gynecology (61.1%), and internal medicine (59.3%) [10]. In emergency departments (EDs), several factors converge that could contribute to WPV incidents. Concerning the patients, alcohol and drug intoxication and psychiatric illnesses were identified as important risk factors [3, 7, 8]. In terms of the organization and staff in EDs, long waiting times for patients, shift work, understaffed EDs, and high job demands of staff increased the risk of WPV [3, 7, 11]. Many emergency physicians believe that the threat of WPV ‘‘comes with the territory’’ of working in the EDs.

Previous studies have been conducted to investigate WPV and its risk factors among HCWs in EDs [3, 12–17]. Specially, studies that have focused on emergency physicians were limited [13, 14, 18]. To date, only one study investigated the prevalence and predictors of WPV toward 1103 frontline clinicians in EDs during the COVID-19 pandemic in China [19]. The lack of national-level data, it is difficult to provide comprehensive information and implement appropriate prevention policies and measures to prevent violence toward physicians in EDs. Therefore, this observational, national, cross-sectional survey aimed to investigate the prevalence of WPV toward emergency physicians and to determine the associated factors of different types of WPV in China.

## Methods

### Study population and sampling

A cross-sectional study was carried out in China from July 2019 to September 2019. The sample size was calculated according to the formula, *n* = [*Z*^2^*π*(1 − *π*)]/δ^2^ (*n* = sample size, *Z* = confidence level for a normal distribution, *π* = expected prevalence, and *δ* = absolute error). The prevalence of WPV among healthcare professionals in EDs in China was 79.8%, which was reported in a previous meta-analysis [10]. Taking a confidence interval (CI) of 95% and an absolute error of 1%, the ideal sample size was 6193. This sample size was increased to 6812 to compensate for nonresponses.

There are 22 provinces, 5 autonomous regions, and 4 municipalities of mainland China (not including Hong Kong, Macao, and Taiwan), and China is usually divided into eastern, central, and western regions. A multistage stratified random sampling design was used in this study. First, a total of 31 Chinese provinces/autonomous regions/municipalities were classified as high-developed, medium-developed, and low-developed regions according to per capita disposable income in 2018 (Additional file 1: Appendix). Second, we selected 10 hospitals randomly from each province/autonomous region/municipality. Third, according to the number and scale of the hospitals, from each sampled hospital, 40% of the emergency physicians who had practiced in the EDs for at least 6 months were randomly selected to complete a self-administered questionnaire. A simple random sampling method was used at each stage. The eligibility criteria of participants were: (1) physicians practiced in EDs; (2) worked for at least 6 months. In total, 15 455 emergency physicians were asked to participate in this survey, and 182 physicians did not respond. In addition, 30 questionnaires were discarded, because information on WPV was missing. Given the purpose of investigating the prevalence of WPV in the past year, we further excluded 395 emergency physicians whose work tenure was less than 1 year. Ultimately, 14 848 eligible questionnaires were used in this analysis. Thus, the sample size was sufficient for our study.

The study protocol was approved by the Institutional Ethics Board of the Second Affiliated Hospital of Hainan Medical University, Haikou, China (HYLL-2018-035). We obtained the consent of each medical institution involved in this study. All individuals provided written informed consent to the researchers before the survey, and participants’ personal information was kept confidential.

### Instrument and measurement

The questionnaire was designed based on literature reviews, group discussions, and preliminary interviews. The questionnaire consisted of six parts: factors associated with WPV (sociodemographic information, health-related factors, and work environment related to WPV prevention), WPV, attitudes toward pre-hospital first aid, job burnout, depression symptoms, and turnover intention. Given the purpose of this study, the data from section 1 and 2 were included.

By reviewing the literature [6, 7, 13, 20, 21], we extracted the factors that might predict WPV: (1) sociodemographic characteristics: region, age, sex, marital status, education level, level of hospital, ownership, work tenure, contract status, professional title, income status, and shift work; (2) health-related factors: body mass index (BMI), history of hypertension, history of diabetes, history of coronary heart disease (CHD), smoking status, and alcohol drinking; (3) work environment related to WPV prevention: whether having preventive measures of WPV, having preventive training of WPV, and encouraging to report WPV. In this study, “shift work” was evaluated by one item “do you have night shifts in your usual work?” with 2 response options (yes/no).

The dependent variable was WPV toward emergency physicians. The Chinese version of the Workplace Violence Scale (WVS), developed by Wang et al. [22], has good reliability and validity for measuring the incidence of WPV when applied to medical staff in China. It includes 5 items measured with a four-point ordinal scale (0 = never, 1 = once, 2 = two or three times, 3 = more than three times). Five types of WPV including physical assault, verbal abuse, threat, verbal sexual harassment, and sexual assault were investigated. The total score of the WVS was created by adding the scores for each item, ranging from 0 to 15, with higher scores indicating a higher frequency of experiencing WPV. Scoring 0 was considered not to have been exposed to WPV, and scoring 1 to 5, 6 to 10, and 11 to 15 indicated low, medium, and high frequency of WPV, respectively [22]. WPV was further classified as physical (physical assault and sexual assault) and nonphysical (verbal abuse, threat, and verbal sexual harassment) violence. In this study, Cronbach’s alpha for WVS was 0.80.

Moreover, the perpetrators, causes, and reactions to the most recent experience of WPV in the past year were assessed in this study. The perpetrators of WPV were investigated by one closed-ended, multiple-choice question with 8 responses (patients, patients’ families, colleagues, managers/supervisors, external colleagues, general public, visitors, and others). Two closed-ended, multiple-choice questions (more than one answer) were used to investigate the reasons for WPV (long waiting times, dissatisfied with GPs’ service, unmet patient needs, perpetrators’ death, perpetrators’ mental disorder, thinking medical costs high, requiring financial compensation, after taking drugs or drinking, dissatisfied with treatment effect, and others) and the reactions to WPV (took no action, tried to pretend it never happened, stopped the perpetrators, told friends or families, told colleagues, sought help from managers, sought help from union, called the police, transferred to another position, completed a WPV report, pursued prosecution, and others), respectively.

### Data collection and quality control

In the stage of study design, we invited 20 healthcare experts to evaluate the content validity of the questionnaire. A pretest involving 50 emergency physicians was conducted in Wuhan’s community hospitals to improve the quality of the questionnaire. A total of 47 respondents could clearly understand all the context of the questionnaire. Further modifications were made according to the feedback of respondents. A web link to the online questionnaire, which was designed using Questionnaire Star, was disseminated to participants through WeChat (similar to WhatsApp in Western countries, WeChat is the largest communication platform in China, with over one billion users). To prevent the same participant from repeatedly answering the questionnaire, each device (e.g., smartphone or computer) was eligible to complete the questionnaire once, and logical checks were concurrently run on the Questionnaire Star platform to identify invalid questionnaires. The data were entered into a web-based database by trained investigators to ensure accuracy.

### Data analysis

The descriptive analyses used means (standard deviation, SD) for the continuous variables and frequency (percentage) for the categorical data. In the logistic regression model, the predictive variables included socioeconomic development level (developed, middle-developed, less-developed), geographic region (eastern China, central China, western China), age (< 45 years, ≥ 45 years), sex (male, female), marital status (unmarried/widowed/divorced, married), education level (associate’s degree or vocational diploma, bachelor degree, master degree or higher), level of hospital (others, secondary hospital, tertiary hospital), ownership (non-governmental, governmental), work tenure (< 10 years, ≥ 10 years), contract status (permanent, temporary), professional title (elementary or below, intermediate, senior), income status (high, middle, low), shift work (yes, no), BMI (< 18.5 kg/m^2^, 18.5–< 24.0 kg/m^2^, 24.0–< 28.0 kg/m^2^, ≥ 28.0 kg/m^2^), history of hypertension (yes, no), history of diabetes (yes, no), history of CHD (yes, no), smoking status (nonsmokers, smokers), alcohol drinking (nondrinkers, drinkers), whether working in hospitals which had preventive measures of WPV (yes, no), had preventive training of WPV (yes, no), or encouraged to report WPV (yes, no). Any type of violence, physical violence, and nonphysical violence, as the dependent variables, were treated as categorical variables. Multivariable stepwise logistic regression analysis (cutoffs for selection and elimination: *P* = 0.05 and *P* = 0.10, respectively) was used to calculate the odds ratios (ORs) and 95% CIs for factors that might be associated with the prevalence of WPV (any type of violence, physical violence, and nonphysical violence) toward emergency physicians. All analyses were performed using Statistical Package for Social Sciences (SPSS, Inc., Chicago, IL, Version 27.0) software, and all tests were two-sided with a significance level of 0.05.

## Results

Table 1 presents the main characteristics of the survey respondents. A total of 14 848 respondents were included in the data analysis, yielding an effective response rate of 96.07%. Among the 14 848 respondents, approximately 40% of respondents were from medium-developed regions. Slightly more than one-third (33.58%) of participants were from western China. The mean age was 37.84 (SD = 8.01) years. Most were men (70.53%), married (84.16%), and had a bachelor’s degree or above (94.19%). More than 65% of the participants had intermediate or senior professional titles. In total, 55.65% of the respondents were in the middle or higher level of income groups, and the majority of the respondents experienced shift work. Less than two-thirds of participants worked in hospitals which had preventive measures for WPV.

**Table 1:** Distribution of any type of WPV, physical and nonphysical violence against emergency physicians

Variables	Total n (%)	Any type of WPV^∗^ n (%)	Physical violence n (%)	Nonphysical violence n (%)
Total	14 848 (100.00)	13 423 (90.40)	7639 (51.45)	13 363 (90.00)
Socioeconomic development level				
High	5084 (34.24)	4558 (33.96)	2414 (31.60)	4536 (33.94)
Medium	5876 (39.57)	5425 (40.42)	3178 (41.60)	5402 (40.43)
Low	3888 (26.19)	3440 (25.63)	2047 (26.80)	3425 (25.63)
Geographic region				
Eastern China	5857 (39.45)	5256 (89.74)	2762 (47.16)	5231 (89.31)
Central China	4005 (26.97)	3686 (92.03)	2210 (55.18)	3676 (91.79)
Western China	4986 (33.58)	4481 (89.87)	2667 (53.49)	4456 (89.37)
Age, years				
< 45	11 451 (77.12)	10 416 (90.96)	5728 (50.02)	10 372 (90.58)
≥ 45	3397 (22.88)	3007 (88.52)	1911 (56.26)	2991 (88.05)
Sex				
Male	10 472 (70.53)	9587 (91.55)	6020 (57.49)	9548 (91.18)
Female	4376 (29.47)	3836 (87.66)	1619 (37.00)	3815 (87.18)
Marital status				
Unmarried/widowed/divorced	2352 (15.84)	2080 (88.44)	1087 (46.22)	2067 (88.88)
Married	12 496 (84.16)	11 343 (90.77)	6552 (52.43)	11 296 (90.40)
Education level				
Associate’s degree or vocational diploma^#^	863 (5.81)	753 (87.25)	459 (53.19)	748 (87.67)
Bachelor degree	10,052 (67.70)	9157 (91.10)	5400 (53.72)	9110 (91.63)
Master degree or higher	3933 (26.49)	3513 (89.32)	1780 (45.26)	3505 (89.12)
Level of hospital				
Others	237 (1.60)	180 (75.95)	100 (42.19)	175 (75.84)
Secondary hospital	4691 (31.59)	4264 (90.90)	2560 (54.57)	4242 (90.43)
Tertiary hospital	9920 (66.81)	8979 (90.51)	4979 (50.19)	8946 (90.18)
Ownership				
Non-governmental	624 (4.20)	552 (88.46)	322 (51.60)	549 (88.98)
Governmental	14 224 (95.80)	12 871 (90.49)	7317 (51.44)	12 814 (90.09)
Work tenure, years				
< 10	9462 (63.73)	8546 (90.32)	4699 (49.66)	8511 (90.95)
≥ 10	5386 (36.27)	4877 (90.55)	2940 (54.59)	4852 (90.09)
Contract status				
Permanent	9514 (64.08)	8584 (90.22)	4879 (51.28)	8550 (90.87)
Temporary	5334 (35.92)	4839 (90.72)	2760 (51.74)	4813 (90.23)
Professional title				
Elementary or below	5053 (34.03)	4484 (88.74)	2415 (47.79)	4459 (88.24)
Intermediate	5799 (39.06)	5386 (92.88)	3102 (53.49)	5367 (92.55)
Senior	3996 (26.91)	3553 (88.91)	2122 (53.10)	3537 (88.51)
Income status				
High	1654 (11.14)	1437 (86.88)	790 (47.76)	1429 (86.40)
Middle	6609 (44.51)	5987 (90.59)	3223 (48.77)	5966 (90.27)
Low	6585 (44.35)	5999 (91.10)	3626 (55.06)	5968 (91.63)
Shift work				
Yes	12 956 (87.26)	11 892 (91.79)	6863 (52.97)	11 844 (91.42)
No	1892 (12.74)	1531 (80.92)	776 (41.01)	1519 (80.29)
BMI, kg/m^2^				
< 18.5	436 (2.94)	376 (86.24)	158 (36.24)	376 (86.24)
18.5–< 24.0	6826 (45.97)	6123 (89.70)	3258 (47.73)	6092 (89.25)
24.0–< 28.0	5682 (38.27)	5184 (91.24)	3144 (55.33)	5164 (91.88)
≥ 28.0	1904 (12.82)	1740 (91.39)	1079 (56.67)	1731 (91.91)
History of hypertension				
No	12 544 (84.48)	11 262 (89.78)	6146 (49.00)	11 209 (89.36)
Yes	2304 (15.52)	2161 (93.79)	1493 (64.80)	2154 (93.49)
History of diabetes				
No	14 256 (96.01)	12 886 (90.39)	7249 (50.85)	12 829 (90.99)
Yes	592 (3.99)	537 (90.71)	390 (65.88)	534 (90.20)
History of CHD				
No	14 378 (96.83)	12 977 (90.26)	7281 (50.64)	12 917 (90.84)
Yes	470 (3.17)	446 (94.89)	358 (76.17)	446 (94.89)
Smoking status				
Nonsmokers	11 926 (80.32)	10 713 (89.83)	5881 (49.31)	10 661 (89.39)
Smokers	2922 (19.68)	2710 (92.74)	1758 (60.16)	2702 (92.47)
Alcohol drinking				
Nondrinkers	11 100 (74.76)	9956 (89.69)	5390 (48.56)	9912 (89.30)
Drinkers	3748 (25.24)	3467 (92.50)	2249 (60.01)	3451 (92.08)
Having preventive measures of WPV				
Yes	8930 (60.14)	7848 (87.88)	4205 (47.09)	7802 (87.37)
No	5918 (39.86)	5575 (94.20)	3434 (58.03)	5561 (94.97)
Having preventive training of WPV				
Yes	4744 (31.95)	4016 (84.65)	2177 (45.89)	3992 (84.15)
No	10 104 (68.05)	9407 (93.10)	5462 (54.06)	9371 (93.75)
Encouraging to report WPV				
Yes	7118 (47.94)	6110 (85.84)	3312 (46.53)	6069 (85.26)
No	7730 (52.06)	7313 (94.61)	4327 (55.98)	7294 (94.36)

BMI: body mass index; CHD: coronary heart disease; WPV: workplace violence

^∗^Includes those who experienced only physical, only nonphysical, or both types of workplace violence

^#^Physicians who had acquired associate’s degree or vocational diploma. An associate’s degree required 3 years of education in college after graduation from senior middle school (grade year 10 to year 12) or 5 years of education in college after graduation from junior middle school (grade year 7 to year 9). A vocational diploma required 2 years of education in vocational school after graduation from senior middle school or 3 years of education in vocational school after graduation from junior middle school

Overall, 90.40% of respondents reported exposure to WPV in the previous 12 months. Verbal aggression (87.25%) was the most common form of violence, followed by threat (71.09%), physical assault (48.24%), verbal sexual harassment (38.13%), and sexual assault (19.37%). In addition, 51.45% and 90.00% reported exposure to physical and nonphysical violence, respectively.

### The perpetrators, reasons for, and responses to exposure to violent acts

Table 2 shows the perpetrators, reasons for WPV, and reactions to WPV, respectively. In general, violence, whether physical or nonphysical, was perpetrated mainly by patients’ families and by patients. More than two-thirds of respondents reported that they had been exposed to any type of WPV by families of patients. “Unmet patient needs”, “After taking drugs or drinking”, and “Long waiting times” were the top three reasons for WPV among emergency physicians.

**Table 2:** Perpetrators, causes, and reactions to exposure to WPV

Variables	Any type of WPV^∗^ n (%)	Physical violence n (%)	Nonphysical violence n (%)
Total	13 423 (100.00)	7639 (100.00)	13 363 (100.00)
Perpetrators			
Patients	1676 (12.49)	1054 (13.80)	1666 (12.47)
Patients' families	9345 (69.62)	5749 (75.26)	9320 (69.74)
Colleagues	60 (0.45)	40 (0.52)	60 (0.45)
Managers/Supervisors	33 (0.25)	19 (0.25)	33 (0.25)
External colleagues	22 (0.16)	13 (0.17)	22 (0.16)
General public	70 (0.52)	48 (0.63)	69 (0.52)
Visitors	151 (1.12)	93 (1.22)	149 (1.12)
Others	2066 (15.39)	623 (8.16)	2044 (15.30)
Reasons for violence			
Long waiting times	5098 (37.98)	3185 (41.69)	5086 (38.06)
Dissatisfied with GPs' service	4231 (31.52)	2655 (34.76)	4219 (31.57)
Unmet patient needs	7915 (58.97)	4929 (64.52)	7902 (59.13)
Perpetrators’ death	2128 (15.85)	1476 (19.32)	2120 (15.86)
Perpetrators’ mental disorder	1519 (11.32)	1024 (13.40)	1514 (11.33)
Thinking medical costs high	4776 (35.58)	3085 (40.38)	4770 (35.70)
Requiring financial compensation	2260 (16.84)	1541 (20.17)	2255 (16.87)
After taking drugs or drinking	5852 (43.60)	3992 (52.26)	5831 (43.64)
Dissatisfied with treatment effect	4339 (32.33)	2774 (36.31)	4335 (32.44)
Others	759 (5.65)	472 (6.18)	756 (5.66)
Reactions to violence			
Took no action	3068 (22.86)	1762 (23.07)	3062 (22.91)
Tried to pretend it never happened	1713 (12.76)	1134 (14.84)	1708 (12.78)
Stopped the perpetrators	1921 (14.31)	535 (7.00)	1901 (14.23)
Told friends or families	1213 (9.04)	730 (9.56)	1211 (9.06)
Told colleagues	2991 (22.28)	1825 (23.89)	2989 (22.37)
Sought help from managers	3745 (27.90)	2443 (31.98)	3732 (27.93)
Sought help from union	649 (4.83)	461 (6.03)	644 (4.82)
Called the police	4634 (34.52)	3459 (45.28)	4614 (34.53)
Transferred to another position	192 (1.43)	164 (2.15)	192 (1.44)
Completed a WPV report	3710 (27.64)	2405 (31.48)	3696 (27.66)
Pursued prosecution	145 (1.08)	119 (1.56)	145 (1.09)
Others	783 (5.83)	418 (5.47)	780 (5.84)

WPV: workplace violence

*Includes those who experienced only physical, only nonphysical, or both types of workplace violence

Concerning the reactions of emergency physicians to physical violence, 3459 of 7639 participants who had been attacked called the police (45.28%), 2443 reported it to the managers (31.98%), and 2405 completed an incident/accident report (31.48%). Concerning the reactions of emergency physicians to nonphysical violence, 4614 of 13 363 participants who had been attacked called the police (34.53%), 3732 reported it to the managers (27.93%), and 3696 completed an incident/accident report (27.66%).

### Factors associated with WPV

The results of the multivariable stepwise logistic regression analysis of the factors associated with the prevalence of any type of violence, physical violence, and nonphysical violence among emergency physicians are shown in Table 3. Socioeconomic development level (medium: OR = 1.20, low: OR = 0.75), sex (female: OR = 0.75), educational level (bachelor degree: OR = 1.31), level of hospital (secondary hospital: OR = 3.14, tertiary hospital: OR = 3.55), professional title (intermediate: OR = 1.45, senior: OR = 1.39), income status (middle: OR = 1.25; low: OR = 1.26), shift work (no: OR = 0.37), history of hypertension (yes: OR = 1.52), history of CHD (yes: OR = 1.64), smokers (OR = 1.20), drinkers (yes: OR = 1.25), worked in hospitals without preventive measures (OR = 1.24) or training (OR = 1.58) of WPV and not encouraging to report WPV (OR = 1.99) were predictors of any type of WPV against emergency physicians. The most significant predictors of physical and nonphysical violence were consistent with those associated with any type of WPV. Moreover, geographic region, age, BMI, and history of diabetes were associated factors for physical violence.

**Table 3:** Logistic stepwise regression analysis for the association with exposure to WPV toward emergency physicians

Variables	Any type of WPV^*^		Physical violence		Nonphysical violence	
	OR (95% CI)	*P*	OR (95% CI)	*P*	OR (95% CI)	*P*
Socioeconomic development level (Ref: high)						
Medium	1.20 (1.04–1.37)	0.011	1.02 (0.91–1.14)	0.780	1.19 (1.04–1.36)	0.011
Low	0.75 (0.65–0.87)	< 0.001	0.88 (0.77–1.01)	0.065	0.77 (0.66–0.88)	< 0.001
Geographic region (Ref: eastern China)						
Central China			1.23 (1.09–1.38)	0.001		
Western China			1.30 (1.16–1.46)	< 0.001		
Age, years (Ref: < 45)						
≥ 45			1.32 (1.19–1.47)	< 0.001		
Sex (Ref: male)						
Female	0.75 (0.66–0.85)	< 0.001	0.50 (0.46–0.54)	< 0.001	0.74 (0.66–0.85)	< 0.001
Education level (Ref: associate’s degree or vocational diploma^#^)						
Bachelor degree	1.31 (1.04–1.64)	0.020	1.14 (0.98–1.32)	0.095	1.29 (1.04–1.62)	0.023
Master degree or higher	1.04 (0.81–1.35)	0.751	0.98 (0.83–1.16)	0.833	1.07 (0.83–1.38)	0.605
Level of hospital (Ref: others)						
Secondary hospital	3.14 (2.24–4.39)	< 0.001	1.53 (1.16–2.02)	0.003	3.35 (2.41–4.65)	< 0.001
Tertiary hospital	3.55 (2.54–4.96)	< 0.001	1.58 (1.20–2.08)	0.001	3.82 (2.76–5.29)	< 0.001
Professional title (Ref: elementary or below)						
Intermediate	1.45 (1.26–1.67)	< 0.001	1.18 (1.08–1.27)	< 0.001	1.45 (1.26–1.66)	< 0.001
Senior	1.39 (1.17–1.64)	< 0.001	1.27 (1.13–1.43)	< 0.001	1.39 (1.18–1.64)	< 0.001
Income status (Ref: high)						
Middle	1.25 (1.05–1.48)	0.014	1.05 (0.93–1.17)	0.436	1.26 (1.06–1.49)	0.010
Low	1.26 (1.05–1.52)	0.014	1.36 (1.20–1.53)	< 0.001	1.25 (1.04–1.50)	0.017
Shift work (Ref: yes)						
No	0.37 (0.32–0.44)	< 0.001	0.52 (0.46–0.58)	< 0.001	0.38 (0.32–0.44)	< 0.001
BMI, kg/m^2^ (Ref: < 18.5)						
18.5–< 24.0			1.32 (1.07–1.63)	0.008		
24.0–< 28.0			1.38 (1.12–1.71)	0.003		
≥ 28.0			1.41 (1.12–1.77)	0.003		
History of hypertension (Ref: no)						
Yes	1.52 (1.26–1.85)	< 0.001	1.39 (1.26–1.54)	< 0.001	1.51 (1.25–1.82)	< 0.001
History of diabetes (Ref: no)						
Yes			1.29 (1.07–1.55)	0.008		
History of CHD (Ref: no)						
Yes	1.64 (1.06–2.52)	0.025	2.22 (1.77–2.78)	< 0.001	1.73 (1.12–2.65)	0.013
Smoking status (Ref: nonsmokers)						
Smokers	1.20 (1.01–1.42)	0.038			1.22 (1.03–1.44)	0.020
Alcohol drinking (Ref: nondrinkers)						
Drinkers	1.25 (1.07–1.47)	0.004	1.17 (1.08–1.28)	< 0.001	1.22 (1.05–1.43)	0.009
Having preventive measures of WPV (Ref: yes)						
No	1.24 (1.07–1.45)	0.005	1.32 (1.21–1.43)	< 0.001	1.27 (1.10–1.48)	0.002
Having preventive training of WPV (Ref: yes)						
No	1.58 (1.38–1.80)	< 0.001	1.12 (1.02–1.22)	0.012	1.54 (1.35–1.76)	< 0.001
Encouraging to report WPV (Ref: yes)						
No	1.99 (1.73–2.30)	< 0.001	1.20 (1.11–1.3)	< 0.001	2.00 (1.75–2.30)	< 0.001

BMI: body mass index; CHD: coronary heart disease; CI: confidence interval; OR: odds ratios; WPV: workplace violence

^∗^Includes those who experienced only physical, only nonphysical, or both types of workplace violence

^#^Physicians who had acquired associate’s degree or vocational diploma. An associate’s degree required 3 years of education in college after graduation from senior middle school (grade year 10 to year 12) or 5 years of education in college after graduation from junior middle school (grade year 7 to year 9). A vocational diploma required 2 years of education in vocational school after graduation from senior middle school or 3 years of education in vocational school after graduation from junior middle school

## Discussion

To our knowledge, this is the first study investigating the prevalence of WPV and the associated factors in Chinese emergency physicians at a national level. We found that the prevalence of nonphysical violence was higher than physical violence against emergency physicians in China. The sex, level of hospital, professional title, income status, shift work, history of hypertension, history of CHD, alcohol drinking, working in hospitals which had preventive measures or training on WPV and encouraged to report WPV were common factors associated with any type of WPV, nonphysical violence, and physical violence.

Violence in the workplace is a common phenomenon in the EDs. The findings revealed that 90.40% of Chinese emergency physicians had been exposed to WPV during the previous 12 months. The percentage of emergency physicians experiencing any type of WPV was greatly higher than the overall prevalence of WPV against Chinese ED clinicians during the COVID-19 pandemic (29.2%) [19]. Similarly, the prevalence of WPV against Chinese HCWs decreased after the COVID-19 pandemic [23, 24]. The improved doctor–patient relationship and doctor–patient trust during the COVID-19 pandemic may interpret the variation to some degree [24]. The prevalence of any type of WPV was higher than that reported for emergency physicians in Turkey (78.1%) [13], Morocco (70%) [25], and the USA (74.9%) [26]. However, nonphysical violence was markedly less prevalent [27, 28]. The prevalence of verbal sexual harassment (38.13%) was also lower than that reported in other countries [12, 15, 29]. The differences might be due, at least in part, to differences in sample size and the participants’ characteristics, including their socioeconomic status, their geographic region, the practice setting, the definitions and time period of WPV, and the policy for reporting WPV in their national context.

There were significant differences in the prevalence of any form of WPV, nonphysical violence, and physical violence according to gender, consistent with previous studies [30–34]. One explanation for the lower risk of threat and assault for women emergency physicians may be that women are more likely to adopt specific personal risk reduction measurements than their male counterparts [33]. The lower risk may be also associated with differences in patient caseload and work arrangements [35]. The gendered embodiment relates to feminine vulnerability and masculine strength and control in the context of physical and sexual violence from men but also to men’s assumed vulnerability to allegations of sexual abuse from women patients. Thus, male and female physicians might utilize previously developed ways of doing gender to establish measures to manage violence in particular settings [36]. Another possible interpretation is that male physicians are more easily influenced by the work-related aspects of the job, but female physicians are affected more by the job interfering with their family life. Finally, other researchers have explained this gender difference by hypothesizing that males have greater exposure to violent patients or that there may be a tendency for males to feel more protective of female staff [37].

In our study, we found that emergency physicians who had higher professional titles were at higher risk of any type of WPV, physical violence, and nonphysical violence. Similar results were found among hospital HCWs in China, which indicated that professional title was associated with WPV [34]. A higher professional level may mean a heavier workload, which may affect the quality of physicians’ service delivery [10]. In general, patients have much higher expectations of higher ranking physicians [33]. Patients tend to seek high-level medical services even for minor, self-limiting conditions. Thus, the heavy patient load increased the work stress of physicians, contributing to long-term overwork, which may lead to rushing, indifference, and disrespect toward patients; this can lead to a poor relationship between physicians and patients [33, 38].

Emergency physicians who did shift work were prone to experience WPV, which was consistent with the previous studies [13, 19, 34, 39]. Several reasons could explain the effects of shift work on WPV, including the shortage of staff on night shifts, staff exhaustion, and the consequent impact on patient satisfaction [34]. In our study, the majority of respondents had night shift work. It is necessary to have more security personnel during the night shifts.

Similar to the results reported in the literature [12, 14, 16, 17, 40, 41], our study showed that violence was mainly perpetrated by patients themselves and by patients’ families. However, Jia et al. [41] scrutinized medical WPV incidents reported online in Chinese hospitals from 2010 to 2020 and revealed that visitors were more likely to cause serious damage and mortality to the victims than family members of patients. Visitors came to hospitals not to consult a doctor but to seek out relevant healthcare personnel they may retaliate with [41]. In addition, unmet patient needs and long waiting times were two important reasons for WPV. This indicated that when individuals were exposed to critical health conditions and waited for long times until seen by a physician, they and their relatives had a high level of stress, feelings of anger and frustration which in turn manifested in the form of violence against physicians [16, 42]. The causes of violence against physicians in Chinese EDs were similar to those reported in previous studies [16, 25, 40, 43]. To address the ED-related factors, hospital managers can work on reducing wait times through better utilization of resources and can introduce a triage system to prioritize patients and identify those who cannot wait long to be seen. In addition, medical institutions should offer appropriate treatment to control medical costs, and enhance preventive measures (e.g., adding security personnel, repairing cameras and alarm systems) to protect workers from violence. As we identified, physicians who worked in hospitals that had WPV training programs had a lower risk of experiencing WPV, which was in accordance with a previous study [40]. This highlights that it is warranted to enhance education programs to train HCWs on how to deal with aggressive persons and protect themselves from violence.

### Strengths and limitations

This is the first investigation of the prevalence of WPV and the relevant determinants among emergency physicians in China. The large sample size significantly increased the statistical power to detect determinates of violence. In addition, as WPV is increasingly prevalent in modern society, the results of our study can not only provide baseline data for violence interventional strategies and program assessment in China but can also provide a reference for international comparisons. Third, the survey was anonymous and self-administered, which probably made respondents provide more valid responses, especially regarding sensitive issues such as sexual harassment in addition to eliminating interviewer bias.

Our study had some limitations. First, this study has a cross-sectional design, and we cannot infer causality from the results. Second, all information was collected from a self-report questionnaire and response bias was, therefore, unavoidable. Third, there may be other potential influencing factors for WPV beyond those we investigated; we thus failed to identify all such factors. Fourth, we did not investigate the characteristics of perpetrators, reasons for taking no action among emergency physicians, and subsequent actions of managers or union regarding WPV against emergency physicians, which need to be further assessed in future studies. Finally, we should acknowledge the possibility of underreporting due to time-consuming incident reporting procedures, fear of stigmatization, or a belief that reporting violent incidents will not lead to any positive changes.

### Implications for research and practice

Further research is needed to examine the impact of violence on job satisfaction, burnout, and retention. Interventional studies that examine the impact of measures or programs on decreasing WPV are also needed. Prevention measures might include developing education and training programs to help emergency physicians better manage WPV. It may also be beneficial to increase public awareness of the magnitude of the phenomenon of WPV against emergency physicians through mass media campaigns.

## Conclusions

This survey suggests that violence occurs frequently in EDs in China. The most prevalent type of violence is verbal aggression, followed by threat, physical assault, sexual harassment, and sexual assault. Patients’ families are the main perpetrators of these incidents. Unmet needs of patients, taking drugs or drinking, and long waiting times are the main reasons for violence. Chinese health authorities should establish measures to prevent WPV in healthcare settings, since WPV could threaten the health care system’s sustainability.

## Supplementary Information

**Additional file 1: Table S1. **Distribution of provinces/autonomous regions/municipalities, per capita disposable income in 2018, socioeconomic development level, and geographic region.

## Data Availability

Data may be made available by contacting the corresponding author.

## Abbreviations

BMI: Body mass index
CHD: Coronary heart disease
CI: Confidence interval
EDs: Emergency departments
HCWs: Healthcare workers
ORs: Odds ratios
SD: Standard deviation
SPSS: Statistical Package for Social Sciences
WPV: Workplace violence
WVS: Workplace Violence Scale
