# Supplementary material for: Prevalence and predictors of workplace violence against emergency physicians in China: a cross-sectional study
Source: Hum Resour Health. 2023 Feb 8;21:8. doi: 10.1186/s12960-022-00784-3 (PMC9907873; doi:10.1186/s12960-022-00784-3)
Supplement: Supplementary file 1 — Additional file 1: Table S1. Distribution of provinces/autonomous regions/municipalities, per capita disposable income in 2018, socioeconomic development level, and geographic region. [file 12960_2022_784_MOESM1_ESM.doc]

**Table 1 Distribution of provinces/autonomous regions/municipalities, per capita disposable income in 2018, socioeconomic development level, and geographic region**

| Provinces/Autonomous regions/Municipalities | Per capita disposable income in 2018 (¥) | Socioeconomic development level | Geographic region |
| --- | --- | --- | --- |
| Shanghai (municipality) | 64182.6 | High | Eastern China |
| Beijing (municipality) | 62361.2 | High | Eastern China |
| Zhejiang | 45839.8 | High | Eastern China |
| Tianjin (municipality) | 39506.1 | High | Eastern China |
| Jiangsu | 38095.8 | High | Eastern China |
| Guangdong | 35809.9 | High | Eastern China |
| Fujian | 32643.9 | High | Eastern China |
| Liaoning | 29701.4 | High | Eastern China |
| Shandong | 29204.6 | High | Eastern China |
| Inner Mongolia Autonomous Region | 28375.7 | High | Western China |
| Chongqing (municipality) | 26385.8 | Medium | Western China |
| Hubei | 25814.5 | Medium | Central China |
| Hunan | 25240.7 | Medium | Central China |
| Hainan | 24579.0 | Medium | Eastern China |
| Jiangxi | 24079.7 | Medium | Central China |
| Anhui | 23983.6 | Medium | Central China |
| Hebei | 23445.7 | Medium | Eastern China |
| Jilin | 22798.4 | Medium | Central China |
| Heilongjiang | 22725.8 | Medium | Central China |
| Shaanxi | 22528.3 | Medium | Western China |
| Sichuan | 22460.6 | Medium | Western China |
| Ningxia Hui Autonomous Region | 22400.4 | Low | Western China |
| Shanxi | 21990.1 | Low | Central China |
| Henan | 21963.5 | Low | Central China |
| Xinjiang Uygur Autonomous Region | 21500.2 | Low | Western China |
| Guangxi Zhuang Autonomous Region | 21485.0 | Low | Western China |
| Qinghai | 20757.3 | Low | Western China |
| Yunnan | 20084.2 | Low | Western China |
| Guizhou | 18430.2 | Low | Western China |
| Gansu | 17488.4 | Low | Western China |
| Tibet Autonomous Region | 17286.1 | Low | Western China |
